# DRAGoM: Classification and Quantification of Noncoding RNA in Metagenomic Data

**DOI:** 10.3389/fgene.2021.669495

**Published:** 2021-05-05

**Authors:** Ben Liu, Sirisha Thippabhotla, Jun Zhang, Cuncong Zhong

**Affiliations:** ^1^Department of Electrical Engineering and Computer Science, The University of Kansas, Lawrence, KS, United States; ^2^Division of Medical Oncology, Department of Internal Medicine, University of Kansas Medical Center, Kansas City, KS, United States; ^3^Department of Cancer Biology, University of Kansas Medical Center, Kansas City, KS, United States; ^4^Bioengineering Program, The University of Kansas, Lawrence, KS, United States; ^5^Center for Computational Biology, The University of Kansas, Lawrence, KS, United States

**Keywords:** metagenomics, noncoding RNA, covariance model, homology search, genome assembly

## Abstract

Noncoding RNAs (ncRNAs) play important regulatory and functional roles in microorganisms, such as regulation of gene expression, signaling, protein synthesis, and RNA processing. Hence, their classification and quantification are central tasks toward the understanding of the function of the microbial community. However, the majority of the current metagenomic sequencing technologies generate short reads, which may contain only a partial secondary structure that complicates ncRNA homology detection. Meanwhile, *de novo* assembly of the metagenomic sequencing data remains challenging for complex communities. To tackle these challenges, we developed a novel algorithm called DRAGoM (Detection of RNA using Assembly Graph from Metagenomic data). DRAGoM first constructs a hybrid graph by merging an assembly string graph and an assembly de Bruijn graph. Then, it classifies paths in the hybrid graph and their constituent readsinto differentncRNA families based on both sequence and structural homology. Our benchmark experiments show that DRAGoMcan improve the performance and robustness over traditional approaches on the classification and quantification of a wide class of ncRNA families.

## Introduction

Noncoding RNAs (ncRNAs) can perform versatile functional roles and their importance in cellular physiology is being increasingly recognized. For example, riboswitch is a class of cis-regulator that locates in the 5′-UTR of its target gene and can alter the expression efficiency of the target through alternating its fold structure upon the binding with molecules such as small metabolites or ion ligands (Tucker and Breaker, 2005; Garst et al., 2011; Breaker, 2018). A different trans-regulatory mechanism was found to be exerted by bacterial small RNAs (sRNAs), which attenuate (in rare cases promote) their target mRNA expressions through sequence complementarity-based binding (in a similar way as eukaryote microRNAs) (Gottesman and Storz, 2011; Storz et al., 2011; Nitzan et al., 2017; Waters et al., 2017). ncRNAs can also catalyze biochemical reactions (ribozymes) (Doherty and Doudna, 2001), as exemplified by the well-known ribosomal RNAs (which catalyze protein synthesis) and group I and II introns (which catalyze the excision of themselves from the transcript) (Adams et al., 2004a,b). With the prevalence of metagenomics (Virgin and Todd, 2011; Huttenhower et al., 2012; Shokralla et al., 2012; Williamson and Yooseph, 2012; Davison et al., 2015; Quince et al., 2017), more microbial genomic sequences, including the previously uncharacterized ones, have been accumulated into public databases. The amazing richness of microbial genomic data renders a great opportunity to study ncRNA. Indeed, the diversity and richness of microbial ncRNA function revealed from analyzing metagenomic data are beyond our existing knowledge (Weinberg et al., 2010; Nawrocki and Eddy, 2013a; Tobar-Tosse et al., 2013; Stav et al., 2019), including many long ncRNA classes such as OLE, GOLLD, and HEARO (Harris and Breaker, 2018). The discoveries underpin the importance of ncRNA functions in bacterial physiology, ecology, and interaction with the environment.

Despite the importance of functional ncRNA, reliable classification and quantification of ncRNA elements from metagenomic sequencing dataremain challenging. Because the function of ncRNA is more determined by its structural fold rather than its primary sequence (except few ncRNA classes such as microRNA, Bartel, 2009; Davis and Hata, 2009; Winter et al., 2009), the homology search of ncRNA often relies on both primary sequence and secondary structure conservation (Klein and Eddy, 2003; Zhang et al., 2006). Both types of information of a given ncRNA family can be complied using stochastic context-free grammar (SCFG) into a covariance model (CM) to facilitate family-level homology detection (Eddy and Durbin, 1994), in a similar idea of using the profile hidden Markov model (HMM) for protein family characterization (Sonnhammer et al., 1997). In the context of metagenomic sequencing data, the short reads (∼100–150 bp) may only contain partial secondary structure information, leading to inferior ncRNA homology search performance. The issue has been partially addressed via the development of the truncated Cocke–Younger–Kasami (trCYK) algorithm for parsing reads with an incomplete secondary structure (Kolbe and Eddy, 2009), but its performance remained lower compared to a homology search with a complete secondary structure. On the other hand, while a natural way to resolve this issue is to reconstruct complete secondary structure information via *de novo* genome assembly, the assembly itself remained fragmentary and incomplete for metagenomic data generated from a complex microbial community (Ghurye et al., 2016; Sczyrba et al., 2017; Breitwieser et al., 2019; Olson et al., 2019). Many ncRNA reads, especially the low-abundance ones, may not be assembled into contigs and cannot be detected in the subsequent homology search stage.

To tackle the challenge of ncRNA homology search from metagenomic sequencing data, we have developed DRAGoM (Detection of RNA using Assembly Graph from Metagenomic data). DRAGoM aligns CM against paths in an assembly graph and classifies the paths and their constituent reads into different ncRNA families based on the alignment. Note that a path in an assembly graph corresponds to a set of overlapping reads, which is more likely to contain complete secondary structure information that facilitates homology detection. Hence, we can expect DRAGoM to outperform the strategy of performing a homology search directly on unassembled reads (subsequently referred to as the “read-based” strategy). On the other hand, using the complete set of paths in the assembly graph without topological simplification (e.g., bubble removal and tip trimming, Bankevich et al., 2012; Simpson and Durbin, 2012; Nurk et al., 2017) and traversal (e.g., as Eulerian paths, Pevzner et al., 2001) is more likely to retain the original metagenome information (such as polymorphism and stain-level sequence variation). As a result, DRAGoM is also expected to rescue many ncRNA reads that cannot be assembled into contigs and to outperform the strategy of performing a homology search on assembled contigs (thereafter referred to as the “assembly-based” strategy).

We have benchmarked DRAGoM with a representative of the read-based strategy (i.e., CMSearch, Nawrocki and Eddy, 2013b), which includes the trCYK algorithm (Kolbe and Eddy, 2009) for detecting incomplete secondary structures, and representatives of the assembly-based strategy (i.e., assembling the metagenomic reads using a string graph assembler SGA, Simpson and Durbin, 2012, or a de Bruijn graph assembler SPAdes, Bankevich et al., 2012; Nurk et al., 2017, followed by searching the resulting contigs using CMSearch). Our benchmark experiment has considered both simulated and real datasets and includes16S rRNA and a large collection of CMs for different ncRNA families registered in Rfam (Nawrocki et al., 2015). We show that DRAGoM has a higher performance compared to the read-based or assembly-based method and demonstrates the most robust performance on ncRNA families with different lengths and conservation levels. Thus, DRAGoM will have potential applications in future metagenomic data analyses, as well as in the functional studies of microbial ncRNAs.

## Materials and Methods

### DRAGoM Algorithm

**FIGURE 1:**
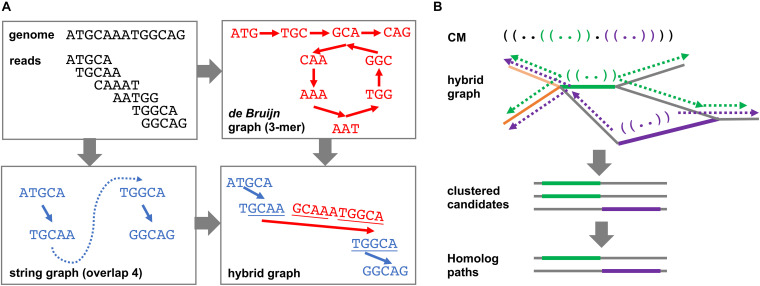
Schematic illustration of the DRAGoM algorithm. **(A)** The construction of the hybrid assembly graph. The hybrid graph, resulting from the merging of a de Bruijn graph and a string graph, can perfectly represent the original genome used in this toy example. **(B)** The search of ncRNA homologs against a hybrid graph. The green and purple parentheses in the querying ncRNACM (covariant model) represent local secondary structural components. The thick green and purple lines in the hybrid graph indicate anchors for path extension. Arrows indicate path extensions of the corresponding anchors.

The DRAGoM algorithm contains two main stages: (1) the construction of a hybrid assembly graph and (2) the identification of homologous ncRNA paths and reads from the resulting hybrid assembly graph. By hybrid assembly graph, we mean the assembly graph resulting from merging a string graph (Myers, 2005) and a de Bruijn graph (Idury and Waterman, 1995), the two main computational models used in sequence assembly. A string graph is constructed based on a suffix–prefix overlap between the reads, while a de Bruijn graph is constructed based on the shared *k*-mers between reads. Either of the model has its own advantages and limitations, with the string graph being more accurate but fragmentary. Both models have been integrated to improve sequence assembly (Huang and Liao, 2016). To illustrate the idea, we present a toy example in Figure 1A. The top-left panel shows an artificial genome sequence and the corresponding short reads. The bottom-left panel shows the string graph constructed from the reads with a minimum overlap length of 4 bp. Because of the uneven (and lower) coverage at the middle of the artificial genome, only four reads out of six can be overlapped. A missing link (the blue dashed line) exists between the two subgraphs, leading to a subsequent fragmentary assembly. For de Bruijn graph construction shown in the top-right panel, all reads can be connected using 3-mers as the vertices. While the de Bruijn graph completely recovers all reads, its graph topology is complex, and it can be traversed in more than one way (with or without going into the loop). However, note that the sequence of one of the traversals (i.e., with the loop) can be aligned to some terminal sequences in the string graph (bottom-right panel, underlined sequences), indicating that the corresponding subgraphs can be reconnected using de Bruijn graph paths. The resulting hybrid assembly graph perfectly represents the original genome.

The hybrid graph construction stage of DRAGoM implements the above intuition. Specifically, SGA (version 0.10.15) (Simpson and Durbin, 2012) was used to generate the string graph, and SPAdes (version 3.13.0) (Nurk et al., 2017) was used to generate the de Bruijn graph. When running SPAdes, the “–meta” tag was enabled to indicate metagenomic input (also known as “metaSPAdes”). Both programs were run in the paired-end mode. Detailed command lines for running both assemblers are available from the Supplementary Methods. The intermediate output of SGA (i.e., the.asqg file) was further simplified (using in-house scripts) to condense unbranched paths into single edges. Terminal edges (i.e., edges with an in-degree or out-degree of 0) of the resulting string graph were then aligned to the set of verified SPAdes contig sequences (no coverage hole, see more in Supplementary Methods) using BWA. Only alignments with a minimum score of 45 (per BWA manual, +1 for a match, −4 for a mismatch, and −6 for a gap), a minimum alignment length of 100, and no clipping at the open end (i.e., the end with a degree of 0 in the string graph) were considered. Then, for each SPAdes contig, if it had recruited more than one alignment, the corresponding terminals in the string graph defined by any pair of alignments were connected using the corresponding interval sequence of the SPAdes contig. If a SPAdes contig had recruited only one alignment, the corresponding string graph terminal was extended using the corresponding prefix or suffix sequence of the contig. SPAdes contigs with no recruited alignment were also retained as isolated vertices in the hybrid graph. In a CAMI (Sczyrba et al., 2017) dataset (DS5 as defined in the **Benchmark Datasets and Metrics** section) that contained ∼15M vertices in its string graph, ∼0.7M connections were made. DRAGoM allows the output of the hybrid graph as its intermediate result, which can be traversed by other assemblers for metagenomere construction.

The second main stage of the DRAGoM algorithm is to identify homologous paths and reads with respect to a given querying CM from the resulting hybrid assembly graph. Intuitively, one can exhaustively enumerate all paths of the hybrid graph and align them against the querying CM. However, this naïve approach would be practically infeasible because the number of paths grows exponentially with the number of reads in the dataset. To address this issue, we designed a filter-based heuristic for the speedup (Figure 1B). To begin with, the querying CM was aligned to each edge of the hybrid graph (note that an edge corresponds to a condensed path without branching, or unitig). The edges bearing significant similarity to the querying CM were recorded as anchors. This stage allowed the detection of conserved short structural components (e.g., the green and purple stem-loops in the CM and the bolded paths in the hybrid graph of Figure 1B). The anchors were then extended toward both directions, aiming to reconstruct complete sequences of the candidate ncRNA homologs (the broken arrows in Figure 1B). The extension lengths for each anchor were determined by length of the unaligned prefix and suffix of the CM (with a further extension of 10% of the prefix or suffix length to account for potential gaps). Because some edges of the hybrid graph might represent similar sequences (e.g., the heavy and light orange edges in Figure 1B), all paths resulting from extending the anchors were subject to sequence redundancy removal using CD-Hit (Li and Godzik, 2006). Finally, the set of nonredundant paths were realigned to the querying CM, and the paths passing the gathering score threshold were selected as homologs of the corresponding ncRNA family. Note that the homologous paths are only being used as templates for classifying individual reads but should not be taken as individual ncRNA genes. This is because many of the homologous paths are derived from the exhaustive traversal of all paths of the graph and could be chimeric and redundant (see more in section **Discussion**). Finally, individual reads were further mapped to the homologous paths for their annotation and to quantify the corresponding ncRNA family in the datasets. More details regarding this stage can be found in Supplementary Methods.

The above algorithm was implemented as the DRAGoM software package. DRAGoM accepts a set of querying CM and a given metagenomic sequencing dataset and assigns a subset of the reads to the corresponding ncRNA families. DRAGoM was implemented using GNU C++ and Python and has been tested under several major Linux distributions (RedHat, Fedora, and Ubuntu). It is freely available under the Creative Commons BY-NC-ND 4.0 License Agreement^1^.

### Benchmark Datasets

We constructed five datasets to benchmark the performance of DRAGoM, as summarized in Table 1. Two datasets were simulated in-house, one was generated by an independent research group for a similar benchmark purpose (Yuan et al., 2015), one was from the open metagenomic data analysis challenge CAMI (Sczyrba et al., 2017), and the last one was from a real human gut microbiome (SRR341583). Detailed information regarding the reference genomes included their respective relative abundances, and the *in silico* simulation parametersare available from Supplementary Table 1. All datasets are also available for download from https://cbb.ittc.ku.edu/DRAGoM.html. These five benchmark datasets include the following:

**TABLE 1:** Summary of experimental datasets.

Dataset	Description	No. of genomes	Abundance	No. of reads	Read length	Error rate
DS1	REAGO	14	Staggered	4.6M	100	1%
DS2	Streptococcus	8	5x	0.6M	100	1%
DS3	Marine	28	5x	3.7M	100	1%
DS4	Human gut	3,499	Staggered	11.2M	74	–
DS5	CAMI	4,679	Staggered	31.3M	100	–

• DS1 (the REAGO dataset): This simulated dataset represented a low-diversity metagenomic dataset that contains microbes from different clades with staggered abundances. The dataset was used in the benchmark experiment of REAGO (Yuan et al., 2015). It was simulated *in silico* with an average read length of 100nt and an expected error rate of 1%, containing 4,653,918 paired-end reads.
• DS2 (the streptococci dataset): This simulated dataset represented a community with highly related microbial genomes from the same genus (e.g., streptococcus). The dataset was simulated *in silico* using eight streptococcus genomes, with an average read length of 100nt and an expected error rate of 1%. This dataset contained 600,000 paired-end reads.
• DS3 (the marine dataset): This dataset represented a subset of microbial metagenome that was often observed from the marine environment. It was simulated from 28 marine genomes with an average read length of 100nt and an expected error rate of 1% and contained 3,700,000 paired-end reads.
• DS4 (the subsampled gut dataset): This dataset represented a real human gut microbiome community (SRR341583). To facilitate the generation of ground-truth homology for the benchmark purpose, we subsampled the dataset via read mapping to a collection of microbial genomes often found in the human gut environment. Only reads that were mapped to the selected reference genomes were retained, leaving 11,228,362 paired-end reads for this dataset.
• DS5 (the subsampled CAMI dataset): This dataset was downloaded from CAMI (Sczyrba et al., 2017), a comprehensive simulated dataset. To focus on the more challenging cases of metagenomics analysis, only reads representing low-coverage genomes (<10X) were selected (via read mapping). This dataset contained 31,311,294 paired-end reads.

### Benchmark Experiment Setup

Given a querying ncRNA family, we define its ground-truth homologs as the reads that were generated or mapped (>60% of their total lengths) to the genomic intervals that were annotated as the ncRNA family by CMSearch (Nawrocki and Eddy, 2013b) (under its default stringency cutoff). The command lines used for ground-truth generation are available from the Supplementary Methods.

Given the ground-truth definition, we defined true positives (TPs) as the homologous reads that were identified by a given method. We defined false positives (FPs) as the nonhomologous reads that were identified and false negatives (FN) as the homologous reads that were not identified. We further defined recall and precision as

$$\text{Recall}=\frac{T\,P}{T\,P+F\,N},\text{Precision}=\frac{T\,P}{T\,P+F\,P}$$

and subsequently *F*-score as

$$F-\text{score}=\frac{2\times \text{Recall}\times \text{Precision}}{\text{Recall}+\text{Precision}}$$

All methods were tested under a series of different stringency cutoffs to generate the receiver operating characteristic (ROC) curve. The ROC curves were extrapolated to the points (recall: 0, precision: 1) and (recall: 1, precision: 0) to calculate the area under the curve (AUC).

We benchmarked our graph-based ncRNA homology search strategy DRAGoM (homology search against assembly graph) with the read-based strategy (homology search against unassembled reads) and the assembly-based strategy (homology search against assembled contigs). For read-based strategy, we chose CMSearch as the representative and refer to it as “CMSearch” thereafter. For assembly-based strategy, we chose SGA (as the representative of string graph assemblers) and SPAdes (as the representative of de Bruijn graph assemblers) and refer to them as “SGA+CMSearch” and “SPAdes+CMSearch,” respectively. Command lines for executing the programs are available in the Supplementary Methods. Each method was benchmarked using different sets of querying ncRNA families (determined based on their presence in the selected reference genomes, details available from Supplementary Table 2). The reported performance corresponded to the unweighted arithmetic mean performance among the sets of querying ncRNA families. Note that the search performances for 16S rRNA were reported individually, given its importance in metagenome taxonomic profiling.

## Results

**FIGURE 2:**
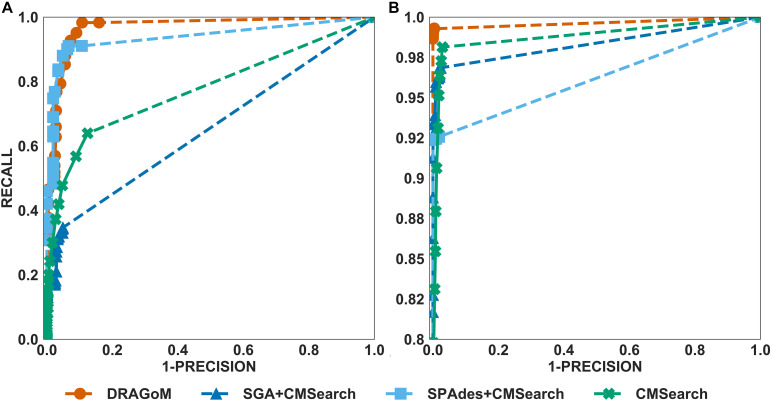
The ROC curves for searching **(A)** 42 non-16S rRNAncRNA families and **(B)**16S rRNAs using the corresponding programs against DS1 (the REAGO dataset).

The performances of all tested methods on DS1 (the REAGO dataset, 42 ncRNA families searched) are shown in Figure 2. For non-16S rRNA queries (Figure 2A), DRAGoM was able to achieve the highest recall, representing a gain of 7.3% recall rate as compared to the second-best performer SPAdes+CMSearch (Table 2). CMSearch alone performed significantly worse than DRAGoM and SPAdes+CMSearch, potentially due to the lack of complete secondary structure information in unassembled reads. SGA+CMSearch seemed to be adversely impacted by the low coverage of this dataset and showed the lowest recall but also showed the highest precision rate. The observation was in line with our current understanding of the characteristics of the string graph and de Bruijn graph assembly approaches. In terms of the peak *F*-score, DRAGoM achieved 93.6%, followed by SPAdes+CMSearch with 92.2%. In terms of AUC, DRAGoM was also the best performer with 96.8%, as compared to 93.9% of the second-best method SPAdes+CMSearch. For 16S rRNA, all methods performed well (Figure 2B). DRAGoM remained the best method with a marginal improvement (99.5% *F*-score and 99.6% AUC, followed by 97.6% *F*-score and 98.8% AUC of the second-best method CMSearch, see Table 3). Surprisingly, SPAdes+CMSearch showed the lowest sensitivity, potentially due to the polymorphism information lost during the graph simplification and traversal stages of SPAdes. Overall, DRAGoM showed a higher performance than any tested method and was robust for both non-16S and 16S rRNA searches.

**TABLE 2:** Performance summary of the tested methods on DS1–DS4 (for non-16S rRNA queries).

Dataset	Matrices	DRAGoM	SGA+CMSearch	SPAdes+CMSearch	CMSearch
DS1	Precision	89.2%	**95.1%**	93.4%	87.6%
	Recall	**98.3%**	34.7%	91.0%	63.9%
	F1	**93.6%**	50.8%	92.2%	73.9%
	AUC	**96.8%**	65.2%	93.9%	77.7%
DS2	Precision	88.7%	**94.0%**	92.0%	91.9%
	Recall	**94.3%**	8.4%	88.4%	49.4%
	F1	**91.4%**	15.5%	90.2%	64.2%
	AUC	**93.0%**	51.3%	90.7%	70.0%
DS3	Precision	87.4%	**93.6%**	91.7%	87.5%
	Recall	**92.4%**	4.6%	87.6%	55.7%
	F1	**89.9%**	8.7%	89.6%	68.0%
	AUC	**94.9%**	49.2%	92.9%	72.9%
DS4	Precision	86.4%	**95.7%**	85.8%	77.6%
	Recall	**65.2%**	23.5%	58.4%	36.9%
	F1	**74.4%**	37.8%	69.5%	50.0%
	AUC	**77.4%**	60.1%	73.3%	58.2%

*The highest performance of each category is bolded.*

**TABLE 3:** Performance summary of the tested methods on DS1–DS5 (for 16S rRNA queries).

Dataset	Matrices	DRAGoM	SGA+ CMSearch	SPAdes+ CMSearch	CMSearch
DS1	Precision	99.9%	99.0%	**100.0%**	97.1%
	Recall	**99.2%**	96.0%	92.4%	98.1%
	F1	**99.5%**	97.5%	96.0%	97.6%
	AUC	**99.6%**	98.4%	96.2%	98.8%
DS2	Precision	96.2%	**98.7%**	97.5%	96.5%
	Recall	**99.9%**	99.7%	97.4%	99.5%
	F1	98.1%	**99.2%**	97.5%	98.0%
	AUC	98.1%	**99.8%**	97.5%	99.4%
DS3	Precision	99.7%	98.6%	**100.0%**	96.8%
	Recall	**98.6%**	94.9%	87.0%	97.7%
	F1	**99.1%**	96.7%	93.0%	97.2%
	AUC	**99.3%**	97.4%	93.4%	98.6%
DS4	Precision	91.9%	97.2%	**97.7%**	97.0%
	Recall	**96.5%**	95.0%	89.8%	79.8%
	F1	94.2%	**96.1%**	93.6%	87.6%
	AUC	94.4%	**96.4%**	94.1%	88.3%
DS5	Precision	**95.1%**	94.7%	94.6%	94.2%
	Recall	**97.7%**	96.9%	92.2%	97.6%
	F1	**96.4%**	95.8%	93.4%	95.9%
	AUC	96.8%	96.6%	94.1%	**97.6%**

*The highest performance of each category is bolded.*

**FIGURE 3:**
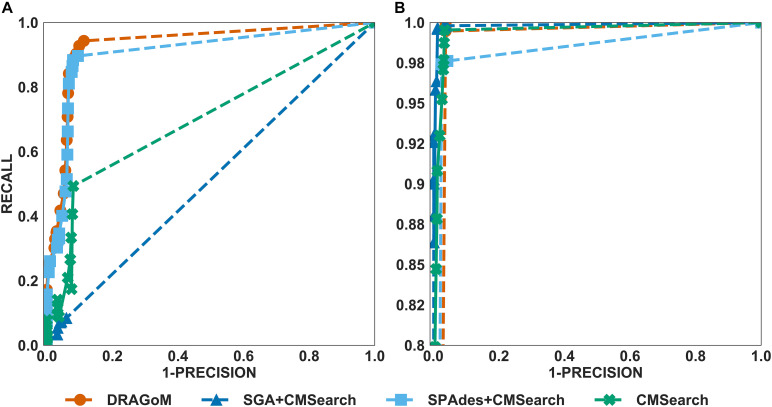
The ROC curves for searching **(A)** 27 non-16S rRNAncRNA families and **(B)**16S rRNAs using the corresponding programs against DS2 (the simulated streptococcus dataset).

For DS2 (the streptococcus dataset, 27 ncRNA families searched), the performance of the methods on non-16S rRNAs was similar to that of DS1 (Figure 3A). DRAGoM again performed the best on this dataset (91.4% *F*-score and 93.0% AUC), followed by SPAdes+CMSearch (90.2% *F*-score and 90.7% AUC, see Table 2). The lower performances of CMSearch and SPAdes+CMSearch were also observed as in DS1 and may be due to similar reasons as discussed previously. For 16S rRNA (Figure 3B), SGA+CMSearch performed the best (99.2% *F*-score and 99.8% AUC), with DRAGoM as the second-best method in *F*-score (98.1%) and CMSearch in AUC (99.4%, see Table 3). The performance of SGA seemed to benefit from its preservation of polymorphism information in 16S rRNA via a more conservative graph simplification strategy. On the other hand, DRAGoM remained the most sensitive method (with the highest recall rate of 99.9%), but its overall performance appeared to be compromised by the lower precision rate due to exhaustive path traversal (96.2%, see Table 3).

**FIGURE 4:**
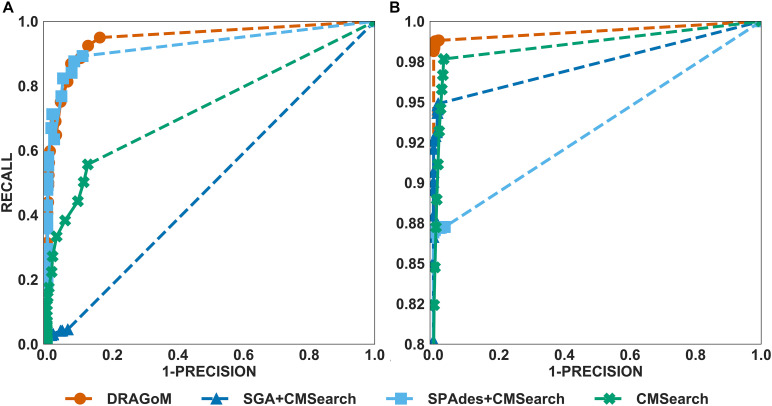
The ROC curves for searching **(A)** 93 non-16S rRNAncRNA families and **(B)**16S rRNAs using the corresponding programs against DS3 (the simulated marine dataset).

**FIGURE 5:**
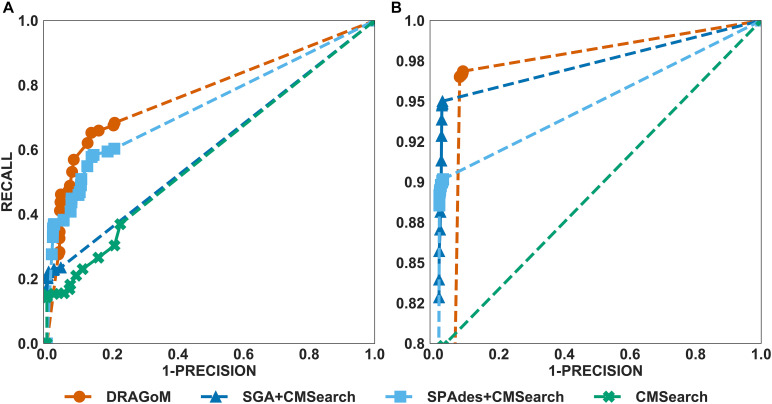
The ROC curves for searching **(A)** 60 non-16S rRNAncRNA families and **(B)**16S rRNAs using the corresponding programs against DS4 (the subsampled human gut dataset).

For DS3 (simulated marine, 93 ncRNA families searched; see Figure 4) and DS4 (subsampled human gut, 60 ncRNA families searched; see Figure 5), the performance of the methods also followed the same trend as that observed in DS1 and DS2. DRAGoM outperformed the other methods in non-16S rRNA queries (for DS3 shown in Figure 4A, DRAGoM had 89.9% *F*-score and 94.9% AUC; for DS4 shown in Figure 5A, it had 74.4% *F*-score and 77.4% AUC). Note that the lower performance on DS4 for all methods was due to the fact that DS4 was generated by subsampling a real dataset, which contains more experimental noises than the simulated ones. SPAdes+CMSearch also remained as the second-best method on both DS3 and DS4. For 16S rRNA, DRAGoM performed the best on DS3 (99.1% *F*-score and 99.3% AUC; see Figure 4B and Table 3). On DS4, SGA+CMSearch performed the best (96.1% *F*-score and 96.4% AUC; see Figure 5B and Table 3), followed by DRAGoM (94.2% *F*-score and 94.4% AUC). These observations were also consistent with those made in DS1 and DS2.

**FIGURE 6:**
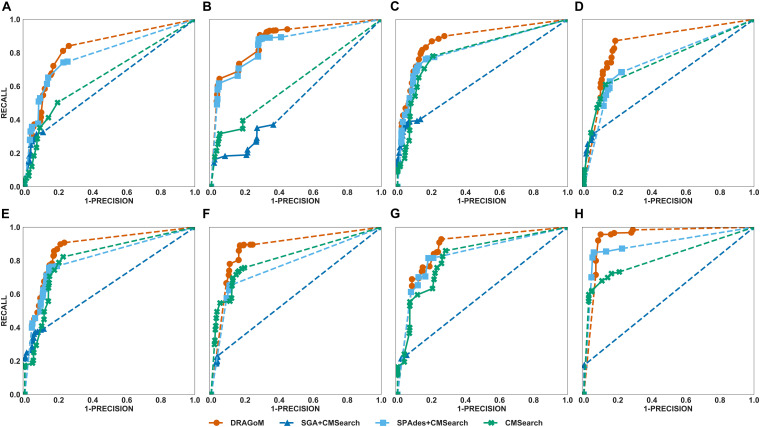
The ROC curves for searching **(A)** 29 ncRNA families with a sequence identity of less than 50% and sequence length from 100 to 200nt, **(B)** eightnc RNA families with a sequence identity of less than 50% and sequence length from 200 to 400nt, **(C)** 78 ncRNA families with a sequence identity from 50 to 70% and sequence length from 100 to 200nt, **(D)** 19 ncRNA families with a sequence identity from 50 to 70% and sequence length from 200 to 400nt, **(E)** 86 ncRNA families with a sequence identity from 70 to 90% and sequence length from 100 to 200nt, **(F)** 19 ncRNA families with a sequence identity from 70 to 90% and sequence length from 200 to 400nt, **(G)** 25 ncRNA families with a sequence identity of more than 90% and sequence length from 100 to 200nt, and **(H)** 12 ncRNA families with a sequence identity of more than 90% and sequence length from 200 to 400nt using the corresponding programs against DS5 (the subsampled CAMI dataset).

DS5 (subsampled CAMI) was tested using the largest number of querying ncRNA families (276); hence, we categorize the performance of non-16S rRNA searches based on the ncRNA families’ sequence identity and average length (Figure 6 and Table 4). Although the performances differed in different categories of ncRNA families, DRAGoM consistently showed the best performance in all categories. The lowest performance gain made by DRAGoM was for the category with<50% sequence identity and 200–400 bp length, where the improvement was 0.6% in *F*-score and 2.4% in AUC compared to the second-best method SPAdes+CMSearch (Figure 6B). The largest gain made by DRAGoM was found in the category with 50–70% sequence identity and 200–400 bp length. Interestingly, the improvement was 11.4% in *F*-score (as compared to SPAdes+CMSearch) and 10.1% in AUC (as compared to CMSearch). Our interpretations for the difference in performance gain in different categories of ncRNA families are present in the **Discussion** section. For 16S rRNA, DRAGoM had the best performance in *F*-score (96.4%, Table 3) but the second-best performance in AUC (96.8%, compared to the best performance of 97.6% made by CMSearch).

**TABLE 4:** Performance summary of the tested methods on DS5 (for non-16S rRNA queries).

Dataset	Matrices	DRAGoM	SGA+CMSearch	SPAdes+CMSearch	CMSearch
Identity:<50%, length: 100–200	Precision	77.5%	**89.2%**	77.3%	80.7%
	Recall	**81.2%**	32.6%	74.3%	50.3%
	F1	**79.3%**	47.8%	75.8%	61.9%
	AUC	**82.4%**	61.7%	78.9%	66.4%
Identity: < 50%, length: 200–400	Precision	71.3%	73.0%	72.1%	**81.4%**
	Recall	**90.7%**	35.2%	87.9%	39.5%
	F1	**79.8%**	47.5%	79.2%	53.2%
	AUC	**87.6%**	52.0%	85.2%	62.2%
Identity: 50–70%, length: 100–200	Precision	85.4%	**86.6%**	83.4%	79.2%
	Recall	**81.8%**	40.4%	76.1%	78.1%
	F1	**83.6%**	55.1%	79.6%	78.6%
	AUC	**87.8%**	65.4%	82.3%	80.7%
Identity: 50–70%, length: 200–400	Precision	81.5%	**94.1%**	78.0%	87.3%
	Recall	**87.2%**	31.8%	68.4%	61.1%
	F1	**84.3%**	47.5%	72.9%	71.9%
	AUC	**85.3%**	63.4%	75.0%	75.2%
Identity: 70–90%, length: 100–200	Precision	82.8%	**88.8%**	85.7%	77.4%
	Recall	**85.7%**	39.3%	75.7%	82.3%
	F1	**84.2%**	54.5%	80.4%	79.8%
	AUC	**87.5%**	65.5%	81.6%	81.6%
Identity: 70–90%, length: 200–400	Precision	83.0%	**96.3%**	88.5%	83.6%
	Recall	**89.1%**	23.0%	65.5%	73.8%
	F1	**86.0%**	37.1%	75.3%	78.4%
	AUC	**87.5%**	59.6%	77.3%	81.7%
Identity:>90%, length: 100–200	Precision	74.2%	**94.6%**	81.8%	71.7%
	Recall	**92.8%**	23.6%	81.5%	85.9%
	F1	**82.5%**	37.8%	81.7%	78.2%
	AUC	**87.7%**	59.4%	83.5%	82.3%
Identity:>90%, length: 200–400	Precision	90.1%	**99.7%**	94.2%	83.8%
	Recall	**95.7%**	17.7%	84.9%	72.3%
	F1	**92.8%**	30.0%	89.3%	77.6%
	AUC	**93.9%**	58.7%	89.5%	81.6%

*The highest performance of each category is bolded.*

Taken together, DRAGoM consistently delivered superior search performance in nearly all datasets and all categories of querying ncRNA families. Specifically, DRAGoM produced the best ncRNA homology prediction for all non-16S rRNA in all datasets and two out five datasets (DS1 and DS3) for 16S rRNA searches (DRAGoM was the second-best method for the other three cases). The assembly-based approach SPAdes+CMSearch seemed to be the second-best choice overall. However, the read-based approach CMSearch appeared to be the second-best choice when analyzing ncRNA families with sequence identity between 70 and 90% and length between 200 and 400 bp (Figure 6F) and in the searches of 16S rRNAs on DS1, DS3, and DS5. Comparably, DRAGoM was the most robust method in addition to its superior performance.

## Discussion

We have demonstrated using benchmark data that DRAGoM can improve ncRNA homology search as compared to the traditional read-based and assembly-based strategies. In addition to the higher performance, another unique advantage of DRAGoM is its robustness. We observed from the benchmark results that the homology search performance is both querydependent and datasetdependent. For example, in DS5 (CAMI), SPAdes+CMSearch performed better than CMSearch when searching ncRNA families with an identity of <50% and between 100 and 200 bp long (Figure 6A) but performed worse than CMSearch for ncRNA families with an identity of 70–90% with the same length range (Figure 6E). We conjecture that some factors could contribute to such a difference. If the ncRNA families are highly divergent, sequence information alone may not be sufficient for its detection, and therefore, the complete secondary structure information needs to be reconstructed for its detection (shown by the higher performance of assembly-based methods for low-identity ncRNA families). On the other hand, for highly conserved families, their corresponding reads could be treated as repeats, with a significant amount of polymorphism information lost (for the lower performance of assembly-based methods for high-identity ncRNA families). Meanwhile, the performance of the existing methods also differs in searching the same ncRNA family against different datasets, as shown by the higher performance of CMSearch (as compared to SGA+CMSearch) in the 16S rRNA search against DS3 (Figure 4B) and its lower performance in the 16S rRNA search against DS4 (Figure 5B). The performance difference could be due to assembly quality. Datasets from less diverse community and sequenced with higher coverage are easier to assemble, leading to the higher performance of assembly-based methods. Given the above observation, the ideal case is that we choose an appropriate analysis strategy based on the query and the dataset. However, it is in many cases infeasible. The robustness of DRAGoM makes it an ideal solution to this issue, allowing consistent biological information to be extracted for diverse research objectives and from heterogenousmetagenomic datasets.

Because DRAGoM directly operates on the assembly graph, the quality of the assembly graph will likely affect the performance of DRAGoM. Currently, the string graph and de Bruijn graph dominate the modeling of sequence overlap information in *de novo* assembly. DRAGoM, which is based on the combination of the two graphical models, outperformed the use of either of them alone (i.e., SGA+CMSearch and SPAdes+CMSearch). The observation is consistent with our current understanding of the two models, where each of them has its unique advantages (where the string graph accurately represents the intact information and the de Bruijn graph generates more complete and longer assembly). We further observed that in most cases, SPAdes+CMSearch outperformed SGA+CMSearch in most cases, suggesting that the reconstruction of a complete secondary structure (facilitated by the longer assembly of SPAdes) is more important than the preservation of polymorphism information (as retained in the string graph). Of course, the conclusion is merely for generic cases, as we did observe examples where SGA+CMSearch outperformed SPAdes+CMSearch (e.g., Figure 5B).

We expect to further improve the speed of DRAGoM in the future. Specifically, the efficiency bottleneck of DRAGoM comes from the fact that it needs to exhaustively align the querying CM with all paths generated from anchors. We envision two potential ways to improve the efficiency, i.e., via more intelligent path filtering criteria and graph simplification techniques. We plan to incorporate additional information, such as the GC content, coverage, and covariant mutation compatibility, to filter out paths that are unlikely to be from the same genome before CM alignment. We also expect to reduce the complexity of the assembly graph through incorporating additional information, such as paired end, long read, or Hi-C data, if applicable (Ghurye and Pop, 2019). In general, we observed that DRAGoM was slower when searching long ncRNA families, because the time for CM alignment and the number of candidate paths to align both grow with the length. As a result, for a long querying ncRNA family, we plan to break it down into a set of smaller components by temporarily removing long-range interactions, aligning each small component individually, and checking if the removed long-range interactions can be recovered given the alignments. This heuristic has been proven effective in speeding up the alignment of RNA structural motifs with pseudo knots while retaining satisfying alignment quality (Zhong et al., 2010). We believe the running time of DRAGoM can be significantly reduced with the above optimization techniques.

In addition to the ncRNA family abundance profile, DRAGoM may also be used to improve taxonomic analysis of metagenomic datasets in two ways. First, DRAGoM can improve the traditional 16S rRNA-based taxonomic analysis. The existing methods for this purpose first identify a set of 16S rRNA-related reads from the metagenomic datasets using read-based homology search, perform local assembly on the identified reads, and then infer the taxonomy (Yuan et al., 2015). DRAGoM can improve this strategy in the 16S rRNA homology search step, as it has demonstrated advantage over the traditional read-based homology search approaches. A more accurate and comprehensive set of 16S rRNA reads to start with before assembly will likely lead to a more complete and finer-grained view of the taxonomic profile, as well as potential insight into the previously unidentified species. A second potential way that DRAGoM can improve taxonomic analysis is through facilitating the use of ncRNA families as taxonomic biomarkers, in a similar way as the protein taxonomic biomarkers (Brocchieri, 2001; Wu and Scott, 2012; Klingenberg et al., 2013). However, we note that in the current implementation, DRAGoM only outputs unassembled homologous reads rather than the assembled ncRNA gene sequences. The reason is that many homologous paths arisen from branchy regions of the assembly graph appear to be artificial and redundant. We plan to incorporate a more sophisticated algorithm into DRAGoM to untangle the homologous paths and to output assembled ncRNA gene sequences, via either finding the minimum set of paths that covers the entire homolog-read assembly graph (as in REAGO, Yuan et al., 2015; and Xander, Wang et al., 2015) or using statistical inference methods that find the most probable subset of paths that explain the observed abundances for each edge (as isoform abundance inference for RNA-seq data, Pertea et al., 2016). We believe that by integrating both protein and ncRNA taxonomic biomarkers, we will be able to obtain unbiased and comprehensive taxonomic profiles.

The current version of DRAGoM only included CMSearch as its core homology search engine, requiring only family-level CMs as queryrather than specific ncRNA sequences. The design is due to the lack of complete reference genomes and concrete gene sequences in many metagenomic studies (Kyrpides et al., 2014). In the future, we plan to further extend DRAGoM to allow for single-sequence ncRNAs as query through providing interfaces for other ncRNA homology search tools. Specifically, we will provide interfaces for RSEARCH (Klein and Eddy, 2003) and FastR (Zhang et al., 2005) if both the ncRNA sequence and secondary structure are available. We will provide interfaces for Dynalign (Mathews and Turner, 2002), FoldAlign (Havgaard et al., 2005), PMcomp (Hofacker et al., 2004), LocARNA (Will et al., 2007), and SPARSE (Will et al., 2015) when only the ncRNA sequence is available. These tools implement variants of the simultaneous alignment and folding (SAF) algorithm (Sankoff, 1985) and do not require an annotated secondary structure for the query. We expect that the incorporation of these software into DRAGoM’s framework will improve the performances by themselves, as DRAGoM provides the hybrid assembly graph and longer candidate paths to characterize the features of different ncRNA genes.

In summary, in this article, we present DRAGoM, a novel algorithm for family-based ncRNA homology search against metagenomic sequencing data. We have demonstrated the advantages of DRAGoM as compared to the traditional read-based and assembly-based approaches.

## Data Availability Statement

The original contributions presented in the study are included in the article/Supplementary Material, further inquiries can be directed to the corresponding author/s.

## Author Contributions

CZ initially conceived the project. CZ and BL designed the algorithm. BL and ST implemented the algorithm. BL performed the benchmark experiments. CZ, BL, and JZ analyzed the results. All authors wrote the manuscript.

## Conflict of Interest

The authors declare that the research was conducted in the absence of any commercial or financial relationships that could be construed as a potential conflict of interest.

## Footnotes

1 https://creativecommons.org/licenses/by-nc-nd/4.0/
