# Supplementary material for: DRAGoM: Classification and Quantification of Noncoding RNA in Metagenomic Data
Source: Front Genet. 2021 May 5;12:669495. doi: 10.3389/fgene.2021.669495 (PMC8131839; doi:10.3389/fgene.2021.669495)
Supplement: Supplementary file 3 [file Table_3.DOCX]

Supplementary Material

# Supplementary Methods

DRAGoM was developed in C++ (g++ v 4.8.5) and python (2.7), containing the following stages:

1. **Generating SPAdes Contigs**

We generated SPAdes (Nurk et al., 2017) (v3.13.0) contigs using the following script:

spades.py --meta -t 16 -m 1024 \

-1 read1.fq -2 read2.fq \

-o spades

For DS3, where the input reads were interleaved paired-end reads, the command line we ran was:

spades.py --meta -t 16 -m 1024 \

--12 read.fq \

-o spades

1. **Generating SGA String Graph**

We generated the SGA (Simpson and Durbin, 2012) (0.10.15) overlap graph using the following steps (as recommended by SGA best practice):

(a) preprocess

sga preprocess -o reads.pp.fastq --pe-mode 1 read1.fq read2.fq

(b) error-correction

sga index --no-reverse -t 16 reads.pp.fastq

(c) remove duplicate reads

sga index -t 16 reads.pp.ec.fa

sga rmdup -t 16 reads.pp.ec.fa

(d) building overlap graph

sga overlap -t 16 -m 30 reads.pp.ec.rmdup.fa

Once the overlap graph was generated, we developed an in-house script to convert the overlap graph into a string graph. The source code of this conversion script is available from the GitHub repository of DRAGoM: https://github.com/benliu5085/DRAGoM.

1. **Generating the Hybrid Graph**

Defile terminal as an edge (i.e., unitig) in the string graph that has either of its ends has a degree of 0; also define open end as the terminal end that has 0 degree. For SPAdes contigs, we further validated them by aligning the individual reads back and only retained the ones that have no coverage hole (i.e., each position in the contig is covered by at least one read). We called these contigs as the trusted contigs. We then align the terminals against the trusted SPAdes contigs using BWA (0.7.16a-r1185-dirty)(Li and Durbin, 2009):

bwa index spades.contigs.fasta

bwa mem -t 16 -a -T 45 spades.contigs.fasta og.fq > og.sam

By using -T 45, we only considered alignments with scores >=45 (per BWA manual, +1 was issued for a match, -4 for a mismatch, and -6 for a gap). We further filter the alignments by ensuring no clipping on the open-end side of the terminal sequence and retaining only alignments that are longer than 100bp. Note that the terminal edges correspond to condensed paths in the string graph, rather than an overlap between two reads as in a traditional overlap graph. In this case, our cutoff of 100bp still applies to datasets with average read length less than 100bp. Once the alignments were collected, we then connect two terminals if they can be aligned to the same SPAdes contig, using the sequence indicated by the corresponding SPAdes contig interval. If the terminal is aligned to a SPAdes contig with no other alignment, the terminal is extended based on the corresponding prefix/suffix sequence of the SPAdes contig. Finally, the SPAdes contigs recruited no alignment were further retained as isolated vertices in the hybrid graph.

1. **Anchoring**

For a given ncRNA query, we used CMsearch (INFERNAL (Nawrocki and Eddy, 2013) 1.1.2) to search the query against each edge of the resulted hybrid graph:

cmsearch -T t1 --nohmmonly --rfam --cpu 16 \

--tblout out.tblout RNA.cm hybrid.graph

All hits reported by CMSearch under its default cutoff were used as anchors.

1. **Generating candidate paths**

For a given ncRNA query, we prioritized the anchors based on their CMSearch E-values. The anchors were then extended towards both directions using a breath-first-search (BFS) algorithm. The extension length is at most 120% of the total length of the prefix or suffix of the querying ncRNA family (based on the anchor position in the ncRNA family), or 100, whichever is smaller. During the BFS, if any lower-priority anchors were traversed, they were subsequently removed from the anchor set and would not be extended again. Finally, the resulted paths were clustered using CD-HIT(Li and Godzik, 2006) (4.7) to remove redundancy:

cd-hit-est -M 0 -c 0.99 -T 16 -o path.cd.fa -i path.fa

1. **Predicting homologous ncRNA reads**

To identify homologous ncRNA paths, we searched the ncRNA query against the candidate paths using CMSearch

cmsearch --cut_ga --nohmmonly --rfam --cpu 16 \

--tblout out.tblout RNA.cm path.cd.fa

We collected the output of the above search as the set of homologous paths (with unaligned flanking sequences trimmed).

As the homologous path set could be large, we performed a two-stage CD-HIT redundancy removal step. In the first stage, redundant (>99% sequence identity) homologous paths are removed. In the second stage, the remaining homolog paths are clustered with 90% sequence identity to avoid missing read mappings due to excessive multi-mapping. Specifically, we ran

cd-hit-est -M 0 -c 0.9 -T 16 -o 90.RNA.cd.fa -i RNA.cd.fa

We then partition the entire homologous path set based on the resulted clustering, such that within each partition no more than 3,000 sequences are from the same cluster. Finally, we aligned the original reads against these partitions using BWA (0.7.16a-r1185-dirty):

bwa index RNA.fa

bwa mem -t 16 -a RNA.fa read.fa

We considered reads that can be aligned to any homologous path with >60% of its total length as the final set of homologous ncRNA reads.

# Experimental Materials

- DS1 (REAGO)

The microbial genomes of this simulated dataset was adopted from REAGO (Yuan et al., 2015). The reference genomes and their relative abundances and accumulated coverages can be found from Supplementary Table 1. The reads were simulated using WGSIM:

wgsim -N2326963 -1100 -2100 -d180 -S7 -e0.01 -r0 \

stagger.reago.fna reago.read1.fq reago.read2.fq

- DS2 (simulated streptococcal genomes)

This dataset was generated using 8 Streptococcus genomes to investigate cases where highly-related genomes are present. The reference genomes and their relative abundances and accumulated coverages can be found from Supplementary Table 1. The reads were simulated using WGSIM:

wgsim -N300000 -1100 -2100 -d180 -S7 -e0.01 -r0 \

strep.fna strep.read1.fq strep.read2.fq

- DS3 (simulated marine)

This simulated data composed of typical microbiomes found in marine. The reference genomes and their relative abundances and accumulated coverages can be found from Supplementary Table 1. The reads were generated using WGSIM:

wgsim -N1850000 -1100 -2100 -d180 -S7 -e0.01 -r0 \

stagger.reago.fna reago.read1.fq reago.read2.fq

- DS4 (subsampled human gut microbiome)

This dataset is a real human gut metagenomic sequencing dataset, downloaded from SRA (SRR341583). The raw reads were first quality-trimmed using Trimmomatic (Bolger et al., 2014) (v0.38):

java -jar trimmomatic-0.38.jar \

PE read1.fastq read2.fastq \

forward.paired.fq.gz forward.unpaired.fq.gz \

reverse.paired.fq.gz reverse.unpaired.fq.gz \

ILLUMINACLIP:TruSeq2-PE.fa:2:30:10 \

LEADING:3 \

TRAILING:3 \

SLIDINGWINDOW:4:15 \

MINLEN:45 -threads 16;

After trimming, only the reads that remained paired after trimming are retained. The reads were then mapped (using BWA) to a set of reference genomes that were often found in the human gut environment (Supplementary Table 1).

bwa index SRR341583.fna

bwa mem -t 16 SRR341583.fna forward.paired.fq reverse.paired.fq

Paired-end reads that has at least one of the ends mapped to the reference genomes were retained, resulted in a total of 11,228,362 reads.

- DS5 (subsampled CAMI)

This simulated data was down-sampled from the CAMI (Sczyrba et al., 2017) toy test dataset labeled with “Medium_Complexity”. The reference sequences (genome, scaffold, and contig) provided by CAMI with designed abundance lower than 10 was selected for the subsampling process (Supplementary Table 1). The reads were quality trimmed using Trimmomatic (v0.38), mapped against the reference genomes using BWA, and subsampled in the same way as DS4. The final dataset contained 31,311,294 read pairs.

Reference

Bolger, A.M., Lohse, M., and Usadel, B. (2014). Trimmomatic: a flexible trimmer for Illumina sequence data. *Bioinformatics* 30(15)**,** 2114-2120.

Li, H., and Durbin, R. (2009). Fast and accurate short read alignment with Burrows–Wheeler transform. *bioinformatics* 25(14)**,** 1754-1760.

Li, W., and Godzik, A. (2006). Cd-hit: a fast program for clustering and comparing large sets of protein or nucleotide sequences. *Bioinformatics* 22(13)**,** 1658-1659.

Nawrocki, E.P., and Eddy, S.R. (2013). Infernal 1.1: 100-fold faster RNA homology searches. *Bioinformatics* 29(22)**,** 2933-2935.

Nurk, S., Meleshko, D., Korobeynikov, A., and Pevzner, P.A. (2017). metaSPAdes: a new versatile metagenomic assembler. *Genome research* 27(5)**,** 824-834.

Sczyrba, A., Hofmann, P., Belmann, P., Koslicki, D., Janssen, S., Dröge, J., et al. (2017). Critical assessment of metagenome interpretation—a benchmark of metagenomics software. *Nature methods* 14(11)**,** 1063-1071.

Simpson, J.T., and Durbin, R. (2012). Efficient de novo assembly of large genomes using compressed data structures. *Genome research* 22(3)**,** 549-556.

Yuan, C., Lei, J., Cole, J., and Sun, Y. (2015). Reconstructing 16S rRNA genes in metagenomic data. *Bioinformatics* 31(12)**,** i35-i43.
